# Leveraging anteroposterior force oscillations to assist walking

**DOI:** 10.1038/s41598-026-47823-w

**Published:** 2026-04-21

**Authors:** Hiva Razavi, Manal Mustafa, Keegan J. Moore, Philippe Malcolm

**Affiliations:** 1 Department of Biomechanics, University of Nebraska at Omaha, Omaha, 68182 USA; 2 School of Kinesiology, University of Michigan, Ann Arbor, 48109 USA; 3 Aircraft Maintenance Department, Amman Arab University, Amman, 11953 Jordan; 4 Daniel Guggenheim School of Aerospace Engineering, Georgia Institute of Technology, Atlanta, 30332 USA

**Keywords:** Engineering, Mathematics and computing

## Abstract

Although humans employ various strategies to minimize metabolic cost, walking still has a substantial cost. Half of this cost comes from leg work to accelerate and decelerate the center of mass (CoM) in the anteroposterior direction. Studies showed how applying forward forces to assist CoM acceleration drastically reduces metabolic cost. However, the effect of combining this with assisting deceleration remains unclear. Here we show that oscillating forward and backward forces with zero net work in total can also reduce the metabolic cost by 11%. In the first part of this manuscript, we used a robotic tether to apply timed forces and found forward forces are most effective when applied around the middle of the double stance (approximately 0–30% of the step cycle), whereas the optimal window for backward forces was broader, spanning roughly 50–80% of the step cycle, and the effect of timing was not strong for this force direction. Nevertheless, the results suggest that a passive device, such as a mass-spring system, could also reduce metabolic cost. In the second part, we describe simulations and experiments of walking with a wheeled mass-spring system. Simulations suggest that certain stiffnesses could reduce the estimated metabolic cost. However, human experiments showed around 60% increase in metabolic cost rather than a reduction, likely due to friction and suboptimal timings. The finding that the results did not match theoretical predictions is similar to challenges in other wearable robotics studies.

**Supplementary Information:**

The online version contains supplementary material available at 10.1038/s41598-026-47823-w.

## Introduction

According to Newton’s first law, an object in motion will stay in a constant state of motion with the same speed and direction unless acted on by an unbalanced force. While walking may seem like a constant state of motion that does not require external forces, it actually does involve accelerations and decelerations that require unbalanced forces and metabolic cost^1^. During each walking stride, the center of mass (CoM) undergoes phases of acceleration and deceleration^2,3^, alternating between inverted pendulum-like vaulting over the stance leg and redirection during step-to-step transitions^4^. The CoM slows to its minimum forward velocity around the middle of the single support phase, after the leading leg applies a braking force (i.e., negative work). It reaches maximum forward velocity near the end of double support, after the trailing leg generates propulsive force (i.e., positive work). Both positive and negative work contribute to the metabolic cost of walking^5,6^. Notably, the acceleration and deceleration of the CoM in the anteroposterior (AP) direction are estimated to contribute 45–47% of the metabolic cost during walking^7,8^.

Reducing metabolic cost can be important, especially for older adults and for those with impaired nervous systems or injuries^9^. Several studies have investigated altering the CoM dynamics to assist walking. In hemiparetic patients, a 10% decrease in vertical CoM displacement was found to reduce metabolic cost by 30%^10^. Several studies have shown how constant forward forces applied at the CoM can reduce the metabolic cost of walking in healthy adults by up to 50%^7,11,12^. More recent studies investigated the effects of non-constant horizontal forces. Bhat et al. employed relatively short and stiff tethers to apply periodic force profiles resulting from forward and backward CoM movements on the treadmill^13^, but found smaller reductions in metabolic cost than those obtained with constant forces. Penke et al. implemented a pulley system that linked the CoM to one of the ankles, enabling the application of cyclic force profiles among poststroke individuals^14^. This system resulted in a 12% reduction in metabolic cost. Our group previously used a robotic tether to investigate the optimal timing and magnitude of forward forces^15^. The greatest reduction in metabolic cost compared to the Zero Force condition was approximately 48%, with the peak timing occurring in the middle of double support at 15% of the step cycle^15^.

Force profiles that alternate between forward and backward directions could, in theory, be applied using an oscillating spring mechanism, enabling the application of forces during walking without the need for a motor. If this is timed optimally, the forward force can assist with acceleration, while the backward force can assist with deceleration. Utilizing a basic pendulum model, Antonellis et al. predicted that a combination of forward and backward impulses with equal magnitudes could reduce metabolic cost by 80%, by entirely eliminating the metabolic cost of accelerating and decelerating the body, such that only the cost of supporting the body weight remains^15^. However, it is essential to note that this was a simplified theoretical prediction that considered walking as an ideal pendulum and assumed that the forward and backward forces were delivered as instantaneous impulses at the very beginning and end of the pendulum movement.

**Fig. 1:**
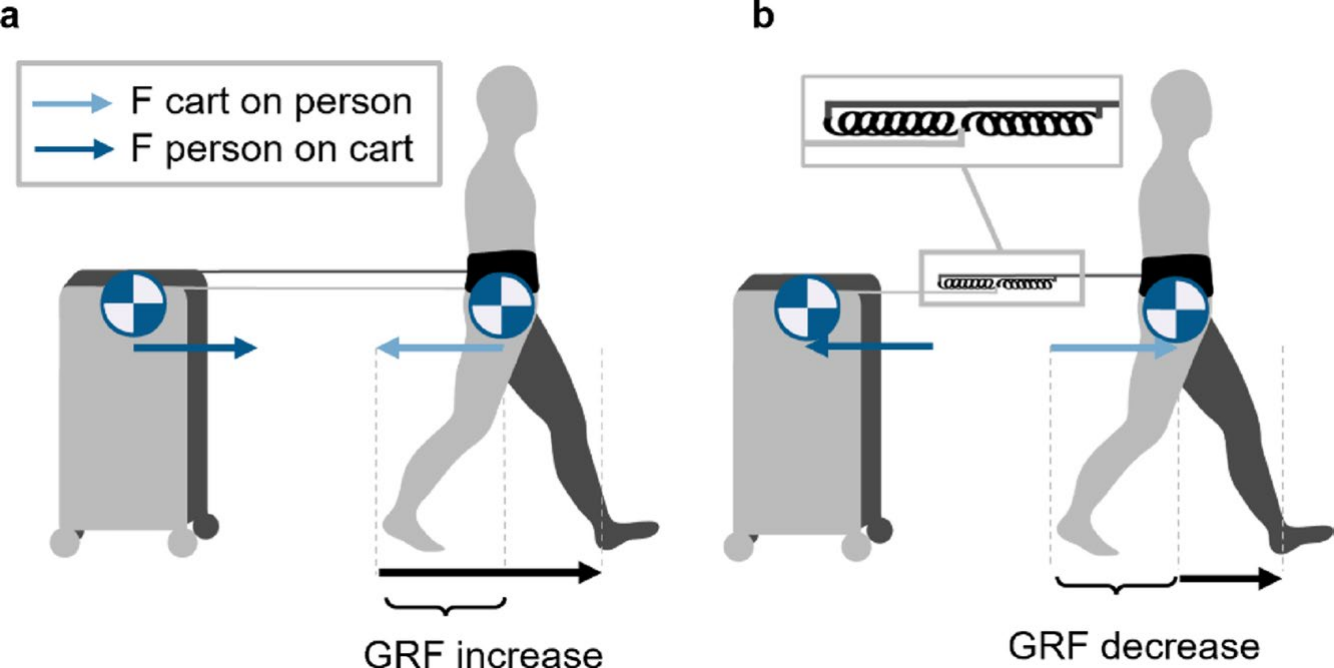
Theoretical mechanism of a mass on wheels. (**A**) A mass is attached to the person by a rigid connection. (**B**) The mass is attached to the person by a bidirectional spring, creating a wheeled mass-spring system. The mass can accelerate in the opposite direction as the COM of the person, thus reducing the ground reaction force (GRF), and thereby the effort and likely also the metabolic cost required by the person to walk.

A mechanism for applying oscillating forward and backward forces in the AP direction could resemble previous work with a vertically oscillating backpack^16^. Carrying added load (e.g., a backpack) increases the mass that must be accelerated and decelerated, leading to increased leg work. Rome et al. reduced the metabolic cost of carrying a backpack by attaching springs and making the backpack mass to oscillates out of phase with the CoM of the wearer^16^. This mechanism reduced the oscillation of the total CoM of the person plus the backpack thereby requiring less work from the legs to produce those oscillations^5^. Other studies of load carriage using compliant bamboo poles also highlighted out-of-phase oscillations between the load and the carrier as a key mechanism for reducing dynamic loading and potentially lowering energetic cost^17,18^. Extending this idea to anteroposterior motion, Li et al. developed an elastic load-carrying device that allowed a load to move forward and backward relative to the body via springs^19^. Although they did not test or report changes in whole-body metabolic cost, their modeling and experiments suggested that spring stiffnesses that result in a resonance frequency below the walking frequency may reduce interaction forces and mechanical work during step transitions. Building on these concepts, connecting a person to a mass on wheels using bidirectional springs with proper stiffness could reduce the CoM oscillations and leg forces (Fig. 1). A fundamental difference compared to the vertically oscillating backpack is that the person would not have to carry added mass. While an oscillating backpack could merely reduce metabolic cost compared to a normal backpack, the mass on wheels could, in theory, reduce metabolic cost compared to walking without added mass. In other words, a passively oscillating mass on wheels could act as an assistive device that makes walking easier.

This manuscript describes two studies aimed at understanding the effect of AP forces applied at the CoM on the metabolic cost during walking. In the first experiment, metabolic cost refers to metabolic energy expenditure derived from indirect calorimetry (i.e., oxygen consumption converted to metabolic power, W·kg⁻¹). In the second experiment, cost of transport was computed as metabolic power normalized by walking speed (J∙(kg∙m)⁻¹).The primary aim was to assess the impact of forward and backward forces with varying timings and magnitudes on the metabolic cost of walking. We evaluated this aim based on statistical analyses of a large dataset recorded from participants walking with cyclical perturbations applied via a robotic waist tether. Based on our group’s earlier research with only forward forces^15^, we expected that applying the forward-directed force in the middle of the double stance phase (15% of the step cycle) would be optimal. Building on the conclusions of our prior work on forward force assistance, we expected that the optimal timing for backward force would not align with peak braking force but rather be related to the inverted pendulum mechanism. Since the CoM spontaneously accelerates due to gravity after reaching midstance, we expected that backward forces between midstance and the end of single stance (i.e., between 65 and 100%) would help control the increase in CoM velocity and thereby minimize the required CoM redirection during double stance. Consequently, we hypothesized that the metabolically optimal timings of peak forward and backward forces are around 15% and 82% of the step cycle, respectively.

The second aim was to evaluate whether the implementation of a mass on wheels connected to a person via a spring could reduce the metabolic cost of walking. To achieve this, we conducted an experiment where participants walked with a wheeled mass-spring system connected to their waist via different bidirectional spring stiffnesses. Participants also walked with a rigid connection (no-spring) as well as without the wheeled mass-spring system (no-cart). We hypothesized that the optimal spring stiffness would reduce metabolic cost compared to walking with no spring connection. We also hypothesized that the mass-spring system would not reduce metabolic cost below the level of walking without a wheeled mass-spring (no-cart). This latter hypothesis is based on the assumption that energy losses due to friction would outweigh the theoretical benefits when compared to walking without a mass.

### Robotic-tether experiment: Evaluation of the feasibility of reducing metabolic cost with forward and backward forces

#### Participants

We evaluated the effects of sinusoidal forces of various magnitudes and timings on the metabolic cost of walking using a within-subjects design. We recruited twelve participants (5 females, 7 males, age: 25.5 ± 2.02 years, weight: 67.0 ± 12.8 kg, height: 1.73 ± 0.08 m, leg length: 0.92 ± 0.06 m) who had no history of musculoskeletal or neurological disorder. The sample size was based on a simplified power analysis (G*power, Germany; sample size for a 1-tailed t-test with 80% power). We assumed an effect size of 0.8 based on the effect size from the largest reduction in metabolic cost from a study with forward forces^15^ and on the assumption that the backward force profiles tested in the present study will likely have four times smaller effects^20^. Participants were recruited using a convenience sampling strategy. Neither participants nor researchers could be blinded to the interventions. However, we ensured that participants remained unaware of any hypotheses related to the optimal conditions. All individuals provided written informed consent prior to participation. This research received approval from the Institutional Review Board of the University of Nebraska Medical Center (UNMC, protocol: 0441-17-FB, approval date: 09/05/17). All methods were performed in accordance with the relevant guidelines and regulations. The person shown in Movie S1 provided consent for his image to be included in an online open access publication.

#### Robotic waist tether

**Fig. 2:**
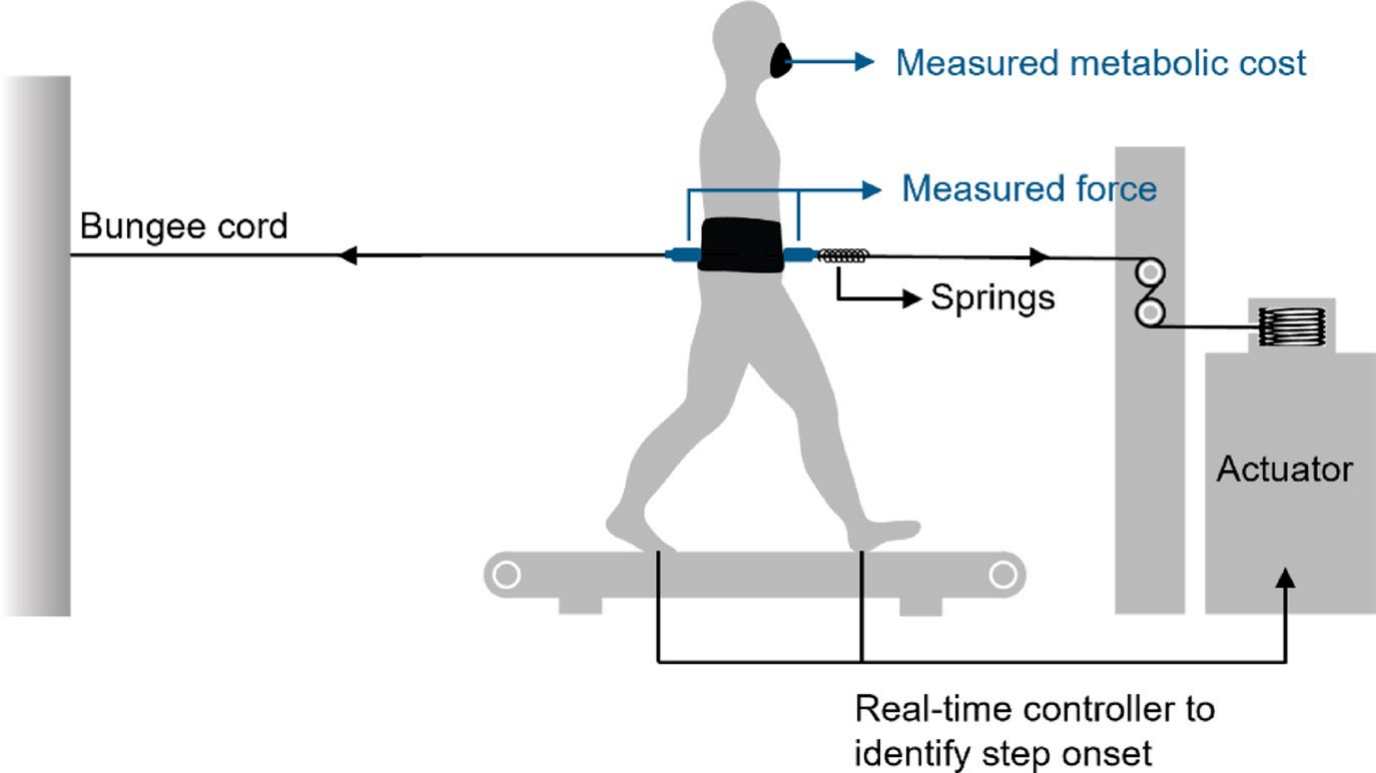
Robotic-tether experiment set-up. The actuator applied the desired force profiles as a function of step cycle percentage in real time. Springs were attached between the front cord and the load cell to help smooth out the applied force and facilitate the force control. A bungee cord connected the participant’s waist to a stable structure located behind them. Load cells connected the waist belt to the cords and measured the actual force being applied. The combined setup of the actuator and the dorsal bungee cord enabled us to apply net forward or backward forces by applying either a greater or smaller force with the actuator relative to the bungee cord.

A cable robot developed based on a commercially available actuation and control platform (HuMoTech, Pittsburgh, PA, USA) allowed the application of desired force profiles to the waist as a function of step cycle percentage (Fig. 2, Movie S1). A motor applied forward forces while a bungee cord applied a nearly constant backward force. By altering the force of the motor, we could produce resultant net resultant forces that were directed either forward or backward. The robot used input from GRFs measured with a force treadmill (Bertec, Columbus, OH, USA) to detect when heel strikes occurred. We developed a high-level controller that enabled us to specify desired force profile shapes (i.e., timing and magnitude, Table S1), and we utilized a low-level controller to adjust the motor output to match the desired force. A similar control system was previously developed by our research group^15^; however, we rewrote it to produce a more simplified version of the controller.

#### Protocol

**Fig. 3:**
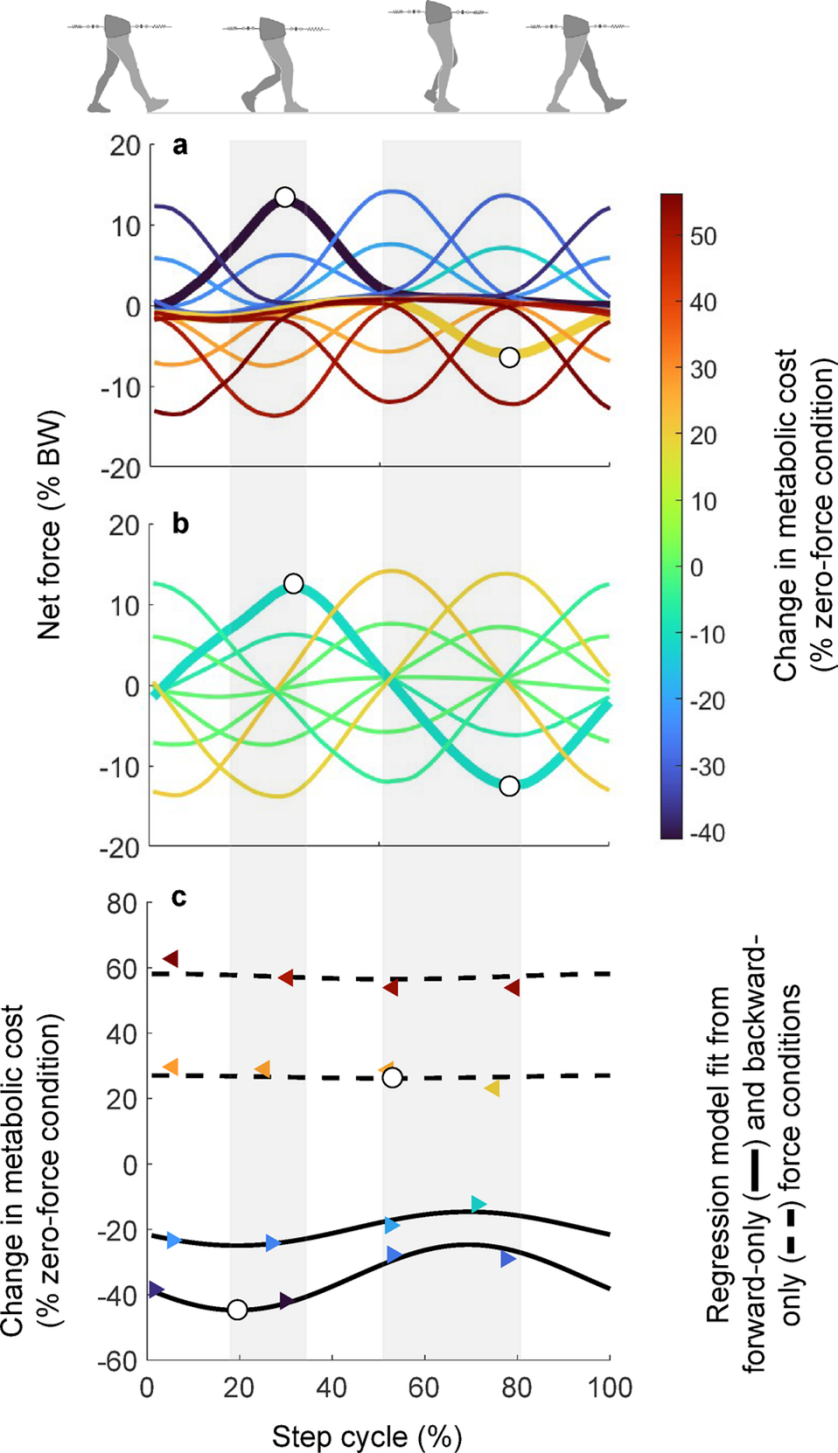
Optimal timings (white dots) from the robotic-tether experiment. (**A**) Optimal timings of robotic-tether peak forces in conditions with only forward or only backward forces, as determined by the greatest reduction (thick blue line) and smallest increase (thick yellow line) in the metabolic cost. (**B**) Optimal timings of robotic-tether peak forces in conditions with both forward and backward forces. (**C**) Regression model fit of the peak positive and negative power timings on metabolic cost across all conditions. Solid black lines show the predicted trend at lower and higher forward-force magnitudes; dashed lines show the trend at lower and higher backward-force magnitudes. Forward- and backward-facing triangles denote individual force conditions, colored by their metabolic change. White circles represent the minimum metabolic cost for each pair of lines. The shaded regions summarize the optimal timing ranges identified under forward-only or backward-only conditions (**a**), under both forward and backward conditions (**b**), and under the regression model (**c**).

To investigate the effects of timing and magnitude of different force profiles, we measured metabolic cost during walking under 26 different conditions. The force profiles included sinusoidal profiles with both a forward and a backward peak in a step cycle as well as profiles with either only a forward or only a backward peak (Fig. 3A, B). Assistance magnitudes ranged from − 15.2% to 15.1% body weight (BW, forward and backward forces are respectively indicated with a positive and negative sign). The duration of all force profiles was 50% of the step cycle. We applied different peak timings over the entire range the step cycle. Participants also walked during a zero-force condition, where the net force was maintained at zero, and a no-tether condition, where they walked without a tether connection. To minimize the effects of learning, participants completed a habituation session where they walked for 1 min in each condition within 2 to 10 days prior to the data collection session, thus completing a total training duration of 26 minuntes^21^. A metronome was used to help isolate the effect of actuation timing by encouraging participants to adhere to a constant step frequency^22,23^. The metronome frequency was chosen based on the participant’s preferred frequency in the zero-force condition in the habituation session. The effects of between-subjects leg length differences were limited by having all participants walk at the same Froude number (0.1)^24^. The resulting mean treadmill speed was 0.95 ± 0.03 m·s^− 1^.

Participants first stood still for 5 min to measure the resting metabolic cost. Each walking condition lasted 2 min, which was sufficient for the instantaneous metabolic cost estimation method^25,26^. The testing protocol was divided into three blocks, with 7-minute rest periods between each block. The sequence of the tethered conditions was randomized except for the zero-force condition, which was repeated twice in the first and last blocks similar to other studies^15,27,28^. The no-tether condition was either done at the very beginning or end of the protocol to minimize setup time losses while also minimizing order effects (Fig. S1).

#### Measurements

We recorded the frontal and dorsal cord forces using load cells (Futek, Irvine, CA, USA) which were filtered with a 10 Hz low-pass Butterworth filter. This cutoff frequency was chosen to be larger than the typical frequency content of walking at ~ 6 Hz^29^ and also based on the bandwidth of the robotic tether^30^. We calculated tether power by multiplying the net resulting tether force by the instantaneous fore-aft CoM velocity. We calculated the instantaneous fore-aft CoM velocity by integrating the CoM acceleration measured with the force plates over time and adding the treadmill speed^31,32^. From the tether power, we determined the actual work, peak power, and timing of the positive and negative power peaks. These measured actuation parameters, which are usually slightly different than the desired settings, were used for further analyses. We also measured GRFs at 2000 Hz using force plates embedded in the treadmill (Bertec, Columbus, OH, USA) and filtered this data in the same way as the load cell forces.

We assessed the metabolic cost using indirect calorimetry (Cosmed K5, Rome, Italy). Recommended calibrations were performed before every session. We converted breath-by-breath measurements to W·kg^− 1^ using the Brockway equation^33^. For the resting trial, the steady-state metabolic cost was calculated by averaging the breath-by-breath data from the last two minutes. For the sinusoidal tether force conditions, the breath-by-breath data, obtained from 2-minute walking trials, were fitted to an exponential function with an individualized time constant that best fit the data. The asymptote from this function then served as the metabolic cost for each condition^25,26^.

#### Statistical analysis

We employed a nonlinear regression model to examine the effects of actuation parameters and to determine the optimal timings for both positive and negative peak powers. The model’s independent variables included the mean waist tether power, the mean waist tether power squared, and the peak timing and magnitude of positive and negative power. Change in metabolic cost compared to the zero-force condition served as the dependent variable. Parameters were chosen based on previous literature with wearable robots that showed the effects of mean power^34^, peak assistance^28^, and timing of assistance^34^. We included second-order terms based on the known U-shaped trend between metabolic cost and forward force magnitude^7,14^. We reduced the number of terms by using net work instead of using positive and negative work separately. The final regression model equation used in the current study was selected after evaluating other candidate equations. The reported R^2^ was computed from the difference between measured and model-predicted metabolic costs, and the p-value was obtained from the Pearson correlation between predicted and observed values (Fig. S2). To identify which conditions produced a significant change in metabolic cost compared to the zero-force condition, we also conducted paired t-tests with a Holm-Šidák correction for multiple testing (*p* < 0.05).

#### Results indicate the feasibility of reducing the metabolic cost of walking with zero net work

The greatest significant reduction in metabolic cost compared with the zero-force condition was 42.8 ± 5.4% (1.15 ± 0.15 W·kg^− 1^, mean ± SEM, *p* < 0.01, paired t-test with Holm-Šidák correction) (Fig. 3a). This reduction occurred in the sinusoidal force condition with only a forward force with a peak of 13.1 ± 0.1% BW and a peak timing at 29.8 ± 0.3% of the step. All conditions with only backward forces increased the metabolic cost compared to the zero-force condition. The smallest increase in metabolic cost resulting from a backward force was 23.9 ± 6.9% (0.57 ± 0.18 W·kg^− 1^) and happened in a condition with a peak of 6.3 ± 0.05% BW at 75.0 ± 3.8% of the step (*p* < 0.01) (Fig. 3a).

When considering conditions that provided both positive and negative work, as in the intended application with a wheeled mass-spring system, the greatest significant reduction in metabolic cost compared to the zero-force condition was only 11.3 ± 5.5% (0.31 ± 0.16 W·kg^− 1^, *p* < 0.05) (Fig. 3b). This reduction occurred in the sinusoidal force condition with forward and backward peak forces of 12.9 ± 0.1 and − 12.8 ± 0.12% BW and peak timings at 31.5 ± 0.5% and 79.3 ± 0.5% of the step. The reduction in metabolic cost despite injecting around zero net work suggests the feasibility of the second aim in this manuscript (assisting with a wheeled mass-spring system).

The regression analysis on all the force profiles led to the following equation for estimating changes in metabolic cost (Eq. (1)):

1 $$\begin{array}{*{20}{l}} \begin{gathered}Metabolic{\text{ }}cost\left( {W\cdot{k}{g^{ - 1}}}\right) =- 3.30 \times {{ \dot W}}aid + 5.05 \times {{ \dot W}}ai{d^2} \hfill \\ -0.28 \times {P_{max}} \cdot sin\left( {\left( {{T_{Pmax}} + 8.86} \right)\div \left( {100 \times 2\pi } \right)} \right) \hfill \\ -0.02 \times {P_{min}} \times sin\left( {\left( {{T_{Pmin}} + 4.18} \right) \div \left( {100 \times 2\pi } \right)} \right) \hfill \\ \end{gathered} \\ {\left( {Adjusted{\text{ }}{R^2}{\text{ }}0.99,{\text{ }}p < 0.001} \right)\;\;\;\;\;\;\;\;\;\;\;\;\;\;\;\;\;\;\;\;\;\;\;\;\;\;\;\;\;\;\;\;\;\;\;\;\;\;\;\;\;\;\;\;\;\;\;\;\;\;\;\;\;\;\;\;\;\;\;\;\;\;\;\;\;\;\;\;\;\;\;\;\;\;\;\;\;\;\;\;\;\;\;\;\;\;\;\;\;\;\;\;\;\;\;\;\;\;\;\;\;\;\;\;} \end{array}$$

Here, ${{\dot W}}aid$ represents the mean waist tether power, while *P*_*max*_, *T*_*Pmax*_, *P*_*min*_, and *T*_*Pmin*_ denote the magnitudes and timings for positive (max) and negative (min) peak powers, respectively. To visualize how peak timing influences metabolic cost in the regression model, we plotted predicted metabolic changes across the range of peak timings for both low and high forward and backward force magnitudes, while keeping other parameters constant at their means (Fig. 3c). This representation showed that the optimal timings for positive and negative peak powers were 19% and 54% of the step cycle, respectively (Fig. 3c). Together with the individual force-profile results, our model identifies a range of optimal timings for forward and backward forces. Forward forces are most effective when applied around the middle of the double stance (approximately 0–30% of the step cycle), whereas the optimal window for backward forces is broader, spanning roughly 50–80% of the step cycle.

### Mass-spring experiment: Implementation in a passive wheeled mass-spring mechanism

#### Preparatory simulation

To prepare the human experiments with a wheeled mass-spring system, we conducted a simplified simulation to estimate the optimal range of spring parameters. The objective was to minimize mechanical effort, quantified from AP GRF and associated work, which served as an indirect estimate of metabolic We performed estimations assuming a subject mass of 70 kg and an oscillating mass of 53 kg. The weight of the oscillating mass was chosen based on the protocol of the human experiments where the cart would weigh 18 kg and we would add half of the participant’s body weight (Fig. 5). We used measured CoM kinematic data from the no tether condition of our first experiment to prescribe the evolution of the human’s CoM position, velocity, and acceleration. We also chose the average speed (~ 1 m/s) and step frequency (~ 1.7 Hz) based on the robotic-tether experiment. We estimated the metabolic cost by dividing the positive and negative portions of the required mechanical work by the person and the corresponding efficiencies^20^. The estimated metabolic cost was calculated under three distinct conditions: walking without additional mass (no-cart), walking with a rigid connection to a mass on wheels (no-spring), and walking with a spring connection to a mass on wheels. The detailed process of the simulation is described in Supplementary Material.

**Fig. 4:**
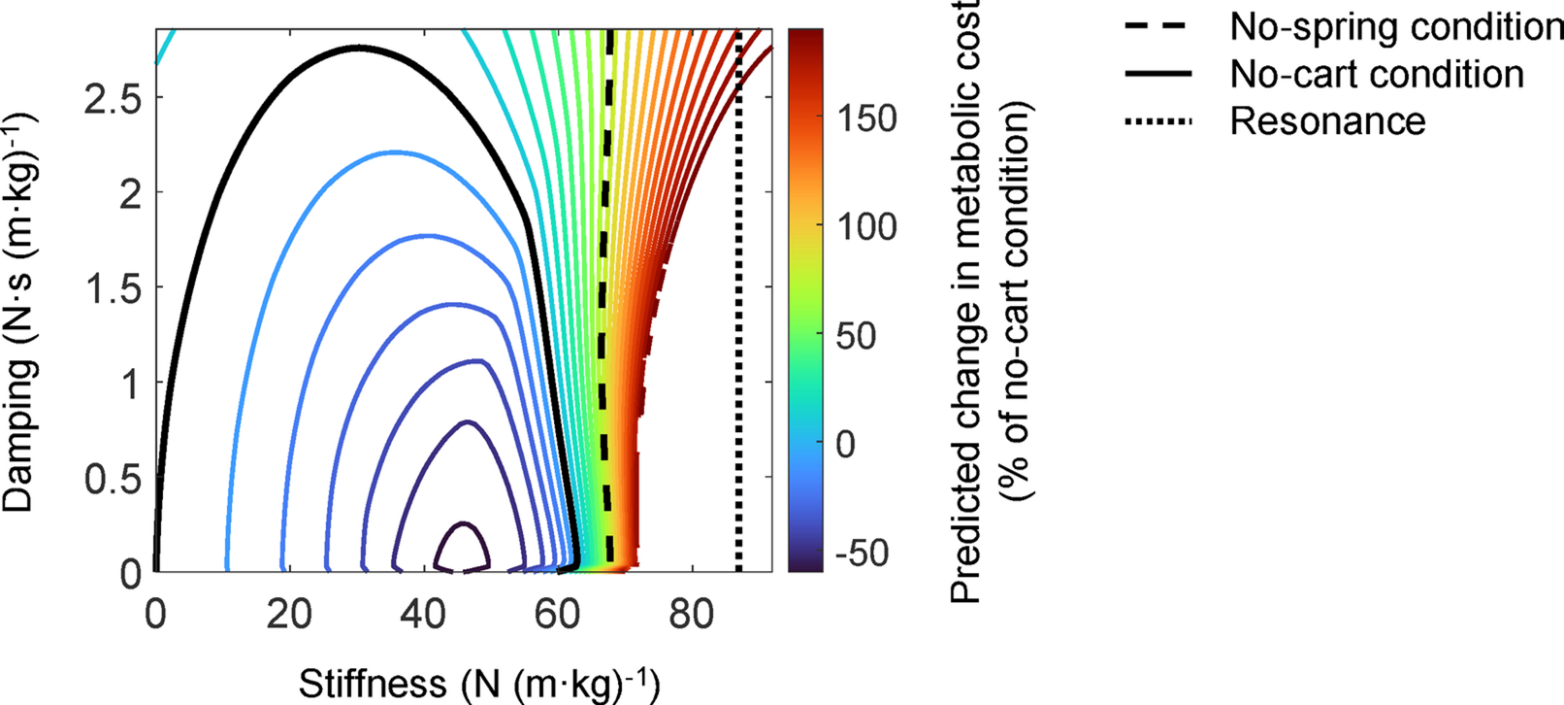
The impact of varying spring and damper constants on estimated metabolic cost, represented by colored lines. The color scale is capped at ± 200% to optimally visualize the region of interest. Dashed and thick black lines indicate where the fitted surface intersects with the magnitude of metabolic cost during a no-cart condition and a no-spring condition, respectively. The dotted line is the stiffness at which the spring–mass resonant frequency would match the walking step frequency.

Our simulations indicated a specific set of spring stiffness and damping constants that are most effective for reducing estimated metabolic cost (Fig. 4). Notably, we observed the greatest reduction in metabolic cost compared to no-cart condition when the spring stiffness was 45.4 N(m·kg)^−1^ combined with a damping constant of 0.03 N·s(m·kg)^−1^. The relative motion between the subject and the wheeled mass–spring system in different regions, and more outputs are shown in Fig. S5 and S6. We speculate that this small amount of damping helps align the timing of the cart’s oscillations with that of the person, since the forward-backward accelerations of the person are not symmetrical in time like a perfect sinusoid. Additionally, the optimal region occurs below the resonance line, where spring–mass resonant frequency would match the walking step frequency (86.9 N(m·kg)^−1^). These results identified a range of spring stiffnesses suitable for the wheeled mass-spring human experiments.

#### Participants

**Fig. 5:**
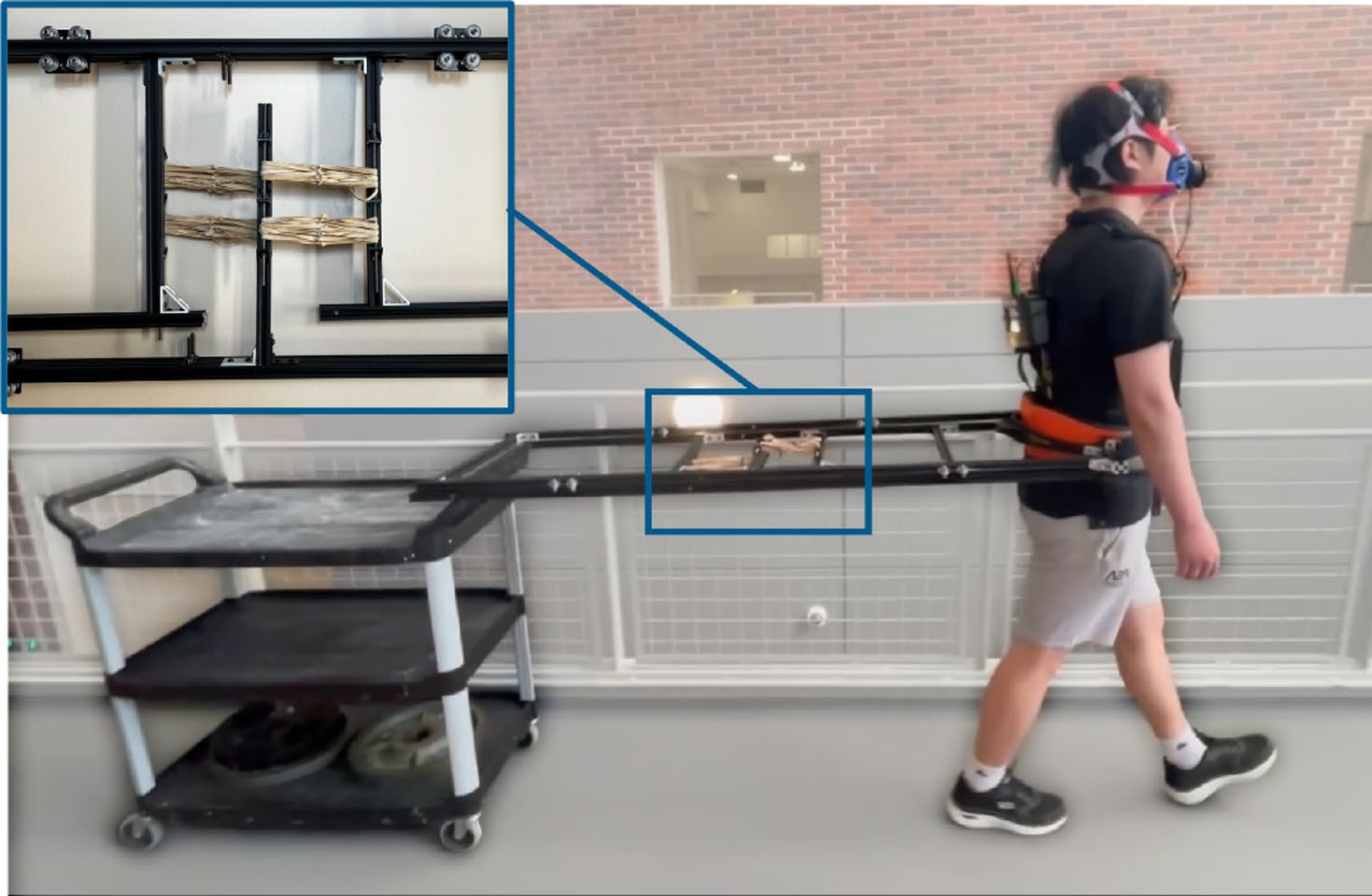
Wheeled mass-spring system. The top and bottom bars were connected to the waist belt and the cart, respectively. These bars were linked by a set of rubber bands and slid over each other along rails in order to act as a bidirectional spring. The participant walked while attached to the wheeled mass-spring by a waist belt. (Picture is modified to improve contrast)

We aimed to test the feasibility of a mass-spring system to reduce the metabolic cost of walking based on the insights learned from the simulation results. We adopted a within-subjects design for this pilot study. We recruited five healthy participants (2 females, 3 males, age: 25.4 ± 2.2 years, weight: 69.3 ± 9.1 kg, height: 1.77 ± 0.04 m). Similar to the robotic-tether experiment, participants were not informed about the hypothesis. All individuals provided written informed consent prior to participation. This research received approval from the Institutional Review Board of the University of Nebraska Medical Center (Protocol: 0050-23-EP, Approval date: 04-03-2023). All methods were performed in accordance with the relevant guidelines and regulations. The person shown in Fig. 5 and Movie S2 provided consent for his image to be included in an online open-access publication.

#### Mass-spring system design

We designed a frame comprising metal bars that could slide on each other using rails (Fig. 5, Movie S2). A pair of antagonistic tension springs (i.e., rubber bands) was mounted into this part. This frame was then attached to a regular cart that could hold the weight plates. The cart, frame, and belt had a total mass of 18 kg, with additional weight plates equal to 50% of the participant’s body weight. This design acted as a bidirectional spring that can both compress and extend relative to its resting position. The frame was attached to a waist belt.

#### Protocol

We evaluated six conditions: springs with four different stiffnesses (lowest: 386, low: 773, high: 1160, and highest: 1547 N·m^− 1^), no-spring (rigid connection), and no-cart (walking with no connections). The spring stiffnesses were relatively lower compared to what was found optimal in the simulation (3400 N·m^− 1^) because of challenges associated with fitting sufficient rubber bands on the device. The spring stiffnesses were measured using a dynamometer. We began with a resting metabolic cost measurement where participants stood still for 5 min. Participants then completed six spring conditions in a randomized order. The data collection took place on a running track (~ 200 m in length with four turns). We asked the participants to walk for 5 min at their preferred walking speed in each condition, with resting periods of at least 5 min between conditions. The participants were instructed to “walk at a sufficiently fast speed” and “try to maintain a constant speed throughout the trial.”

#### Measurements

We employed an odometer-equipped wheel sensor to quantify the walking distance during each 5-minute walking trial. The cost of transport was measured using indirect calorimetry using the K5 (Cosmed, Rome, Italy). The steady-state metabolic cost of transport for each condition was determined by averaging the last two minutes of the breath-by-breath data and standardizing by walking distance.

#### Statistical analysis

We conducted a statistical analysis using a mixed-effects model combined with stepwise regression, where stiffness terms (both linear and quadratic) were included as fixed effects, and the subject numbers were included as a random effect in order to identify significant predictors of the metabolic cost of transport associated with the mass-spring system. We performed the analysis in MATLAB (MathWorks, Natick, MA, USA). The stiffness data included five levels (386, 773, 1160, 1547 N·m^− 1,^ and a rigid condition (no-spring) assumed to have a stiffness of ~ 6000 N·m^− 1^). The 6000 N·m^− 1^ value was arbitrarily chosen, as increases beyond 3000 N·m^− 1^ had no measurable effect on the results. We also compared all conditions using repeated measures ANOVA. We normalized the metabolic cost data by subtracting the baseline (no-cart) condition and expressing it as a percentage of the baseline.

#### Metabolic cost results from the wheeled mass-spring experiment did not match the robotic-tether’s results

**Fig. 6:**
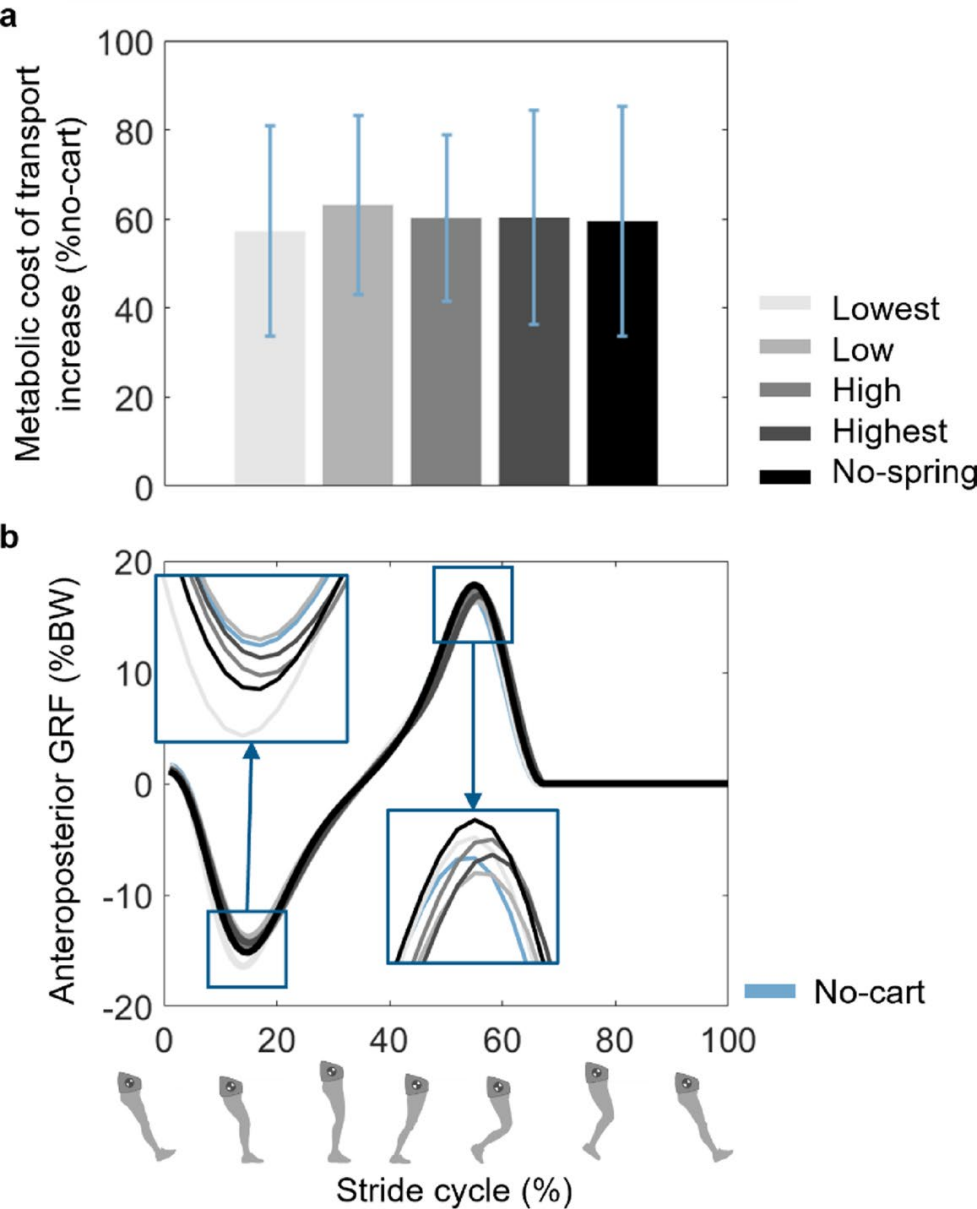
(**A**) Percentage change in the cost of transport compared to no-cart walking. Light blue lines represent SD. (**B**) AP GRF of a participant walking on the treadmill with different spring conditions.

The mixed-effects model analysis did not reveal any significant predictors of the cost of transport. Despite testing the quadratic and linear effects of stiffness, none of the terms reached statistical significance, and the mixed-effects model analysis did not identify significant differences between the stiffness levels. All conditions indicated a significant increase in the cost of transport compared to the no-cart condition (repeated measures ANOVA, *p* < 0.05) (Fig. 6a). The results indicate high variability in metabolic cost across conditions.

The lack of significant findings suggests that the use of a spring-tether, as designed and tested in this study, did not significantly impact the metabolic cost of walking in healthy participants.

## Discussion

This manuscript aimed to evaluate the effect of forward and backward forces with different timings and magnitudes in reducing the metabolic cost and whether a wheeled mass-spring system could also reduce the metabolic cost. We hypothesized that forward and backward forces around 15 and 82% of the step cycle, respectively, would be optimal for reducing metabolic cost. The identified optimal peak timings (19 and 54%) confirmed that forward forces are most effective when applied around the middle of the double stance, whereas the optimal window for backward forces was 50–80% of the step cycle. For the second human experiment, we hypothesized that a wheeled mass-spring system would reduce metabolic costs compared to the no-spring condition. This was not supported by our results.

The effects of assistance magnitude in the robotic tether experiment aligned well with earlier research. Our group’s previous study assessing the effects of different timings and magnitudes of only forward forces found a 47.8% metabolic cost reduction with 15% BW^15^. It may seem like in the current study, the most significant metabolic cost reduction of 42.8% with a force of 13.1% BW was relatively low compared to the previous study^15^. However, we used lower treadmill speeds of ~ 0.95 compared to 1.26 m·s^− 115^. This resulted in a lower amount of work being applied by the tether (as a consequence of a lower product of force and treadmill speed). The slope coefficient of 3.30 of the positive work rate term in the regression equation (Eq. (1)) aligned relatively well with results from foundational studies that showed an efficiency ratio of a 4 W change in metabolic rate per 1 W of mechanical work^20^.

The effects of timing also aligned with previous work and hypotheses from our group. The optimal positive power timing at 19% of the step cycle was close to our previous work, which identified 15% of the step cycle to be optimal^15^. When expressed in stride coordinates, 19% of the step cycle corresponds to approximately 9.5% and 59.5% of the stride cycle. The latter aligns closely with user-preferred assistance timing reported in prior work (54.1–59.2% of the stride cycle)^35^.The optimal timing for the negative peak power, at 54% of the step cycle, was earlier than expected from the hypothesis. However, the effect of timing was relatively weak in backward forces due to the amplitude of the fit (Eq. 1). Backward forces alone could not reduce metabolic cost, although combining backward and forward forces could.

Surprisingly, even backward forces that occurred entirely during the braking phase did not reduce metabolic cost. There could be several reasons for this observation. First, the metabolic efficiency of producing negative mechanical work is substantially higher (approximately fourfold) than that of producing positive mechanical work, meaning that reductions in braking effort yield much smaller metabolic savings than reductions in propulsive effort. Second, the applied braking forces rose much more slowly (~ 25% of the step cycle) than the human braking GRF (~ 13%). It is possible that this less impulsive assistance may be harder for users to exploit effectively. We performed an additional biomechanical analysis of joint work and muscle activation across conditions (Fig. S3). All forward-force conditions reduced both positive and negative joint work and EMG, whereas none of the backward-force conditions showed similar reductions. This suggests that backward forces may influence gait through more complex mechanisms than forward forces, and warrant deeper analysis.

To explore whether backward pulls induced co-contraction, we computed the mean co-contraction index (CCI) over the step cycle using normalized EMG from hip (gluteus maximus, rectus femoris), knee (vastus medialis, biceps femoris), and ankle (tibialis anterior, medial gastrocnemius) muscle pairs (Fig. S4). We found that backward forces, especially in the optimal window identified for backward forces (50–80%) tended to increase ankle co-contraction (Fig. S4c), potentially increasing overall metabolic cost. Interestingly, some backward forces timings appeared to slightly reduce co-contraction in the hip and knee (Fig. S4a, b), perhaps as a result of slowing down the person before leading leg heel strike. Finally, it is possible that humans are less accustomed to backward-directed perturbations, and the brief ~ 2-minute familiarization per condition may have limited the ability to adopt efficient strategies. Together, these factors likely explain why backward braking-phase forces failed to produce metabolic benefits.

Combining a forward force at 31.5% and a backward force at 79.3% of the step cycle reduced metabolic cost by ~ 11%. This result is slightly smaller than what would be expected based on the sum of the changes from the forward and backward components of this combined force profile (i.e., − 41% change in metabolic cost for forward forces at 29% of the step cycle and + 20% change in metabolic cost for backward forces at 78% of the step cycle). The finding that the combined effects still could reduce metabolic cost shows promise for assisting walking with a passive oscillating system that alternates forward and backward forces, such as a wheeled mass-spring system.

The hypothesis of our second aim was not supported. Although all the spring conditions increased the metabolic cost of walking compared to walking without additional mass (no-cart), none of the wheeled mass-spring system conditions reduced metabolic cost compared to the no-spring condition. This was also contrary to our simulation, which suggested that a wide range of stiffnesses could be effective. The average increase in metabolic cost compared to the no-cart condition was 60.7 ± 20.4%. This increase was roughly similar to the percent added mass from the cart (the cart hardware plus the added weights inside the cart amounted to 76 ± 3% BW). As such, this increase was close to results from a previous study that found 50% increase in metabolic power by adding a 50% body mass vest supported by a bodyweight support system^36^.

Several device limitations may explain the relatively large increases in metabolic cost compared to the no-cart condition. The rails connecting the participant to the wheeled mass-spring system restricted arm motion, and this arm motion is important for metabolically economic walking^37^. While the sliding rail mechanism allowed anteroposterior displacements between the wheeled mass and the participant, it restricted the vertical and sideways movement, both of which are known to affect metabolic cost^1,38^. When walking with the cart attached, centripetal forces made turns noticeably more challenging than when walking without the cart. Inspection of video recordings revealed that wheel friction resulted in consistent backward forces, rather than the intended forward and backward oscillating forces. However, these device limitations still do not explain why we did not see reductions in metabolic cost compared to the no-spring condition, since those same limitations existed in that condition. Future designs could also incorporate stiffer or more easily adjustable spring elements, such as coil springs, to enable testing of a broader range of stiffness values, including those that yield resonant frequencies closer to walking step frequencies.

In an attempt to understand how the springs affected the kinetic demands for participants, we performed additional GRF measurements. We measured AP GRF from one participant (male, 29 years, 85 kg, 1.81 m) who walked on a force-measuring treadmill (Bertec, Columbus, OH, USA) while connected to the wheeled mass-spring system, which was set on the ground behind the treadmill. Although this has limitations, the rationale for this setup was that it would eliminate the effects of turns and friction associated with walking on an overground track. This setup was intended to provide insights into the potential effects of forward and backward acceleration of the mass-spring system on the participant’s GRF. We also measured GRF in a no-cart condition.

This additional case study showed that the AP GRF peaks did not increase by 50% compared to no-cart condition, as would be expected from the wheeled mass-spring system (Fig. 6b). This may suggest that the participant reduced his CoM acceleration and deceleration rather than maintaining constant kinematics, which was the basis for the assumption that adding springs would help. We did not observe a clear trend of changes in AP GRF versus spring stiffness. The metabolic cost was not measured during the treadmill case, preventing direct comparison of energetic responses between treadmill and overground conditions.

**Fig. 7:**
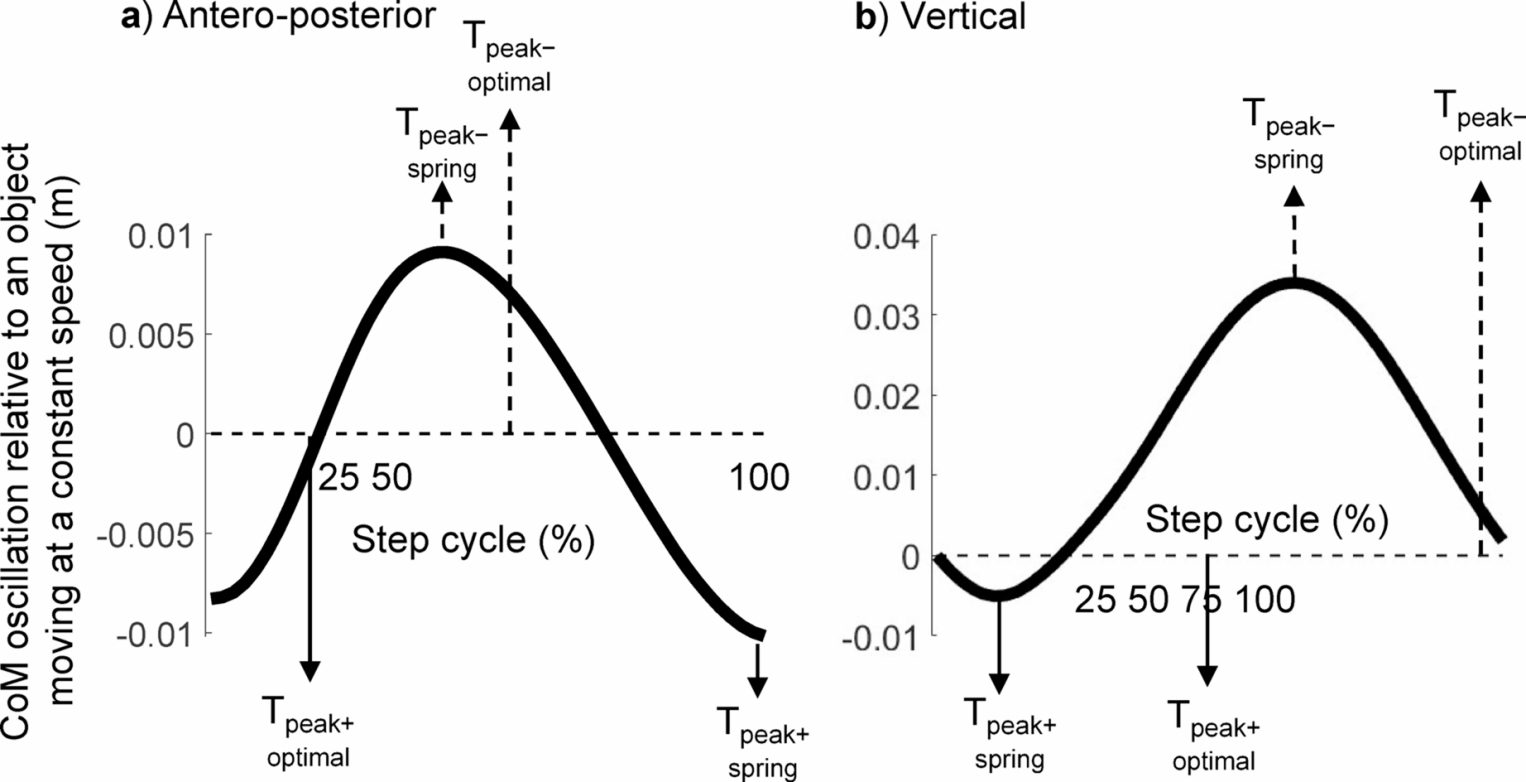
Center of Mass (CoM) oscillation relative to an object moving at constant velocity across a step cycle. (adapted from^15^ during normal treadmill walking). (**A**) AP CoM oscillation during treadmill walking. T_peak−spring_ and T_peak+spring_ are timings when the peak forces of the mass-spring system are likely to occur, which are at the positive and negative peak of the CoM oscillation curve (100% and 42% of a step, respectively). T_peak+optimal_ and T_peak−optimal_ values are predicted based on regression analysis conducted in the current study (19% and 54% of a step, respectively). (**B**) Vertical CoM oscillation during treadmill walking. T_peak−spring_ and T_peak+spring_ values are again identified at the peaks of the CoM curve (11% and 64% of a step). T_peak+optimal_ and T_peak−optimal_ timings are based on findings from Schroeder et al.^23^, with T_peak+optimal_ representing the time point when minimum metabolic power occurred with active oscillations pulling up on the body (after trailing limb toe-off at ~ 43% of the step cycle). T_peak−optimal_ (93%) was estimated based on extrapolating net metabolic power during walking with an oscillator system^23^.

Combining the results from the two parts of this manuscript suggests that the limited benefits could be explained by differences between optimal peak-force timings and the timings when a wheeled mass-spring would apply peak forces. Results from the robotic-tether experiments showed optimal timings for forward and backward forces at 19 and 54% of the step cycle, respectively. Comparing this to changes in AP velocity of the CoM during walking suggests that a wheeled mass-spring system would oscillate with an error of 11% and 19% compared to the optimal timing (Fig. 7a). Similar differences between the optimal and actual timing of systems that oscillate in the vertical direction exist, but they still reduce the metabolic cost (Fig. 7b).

The present manuscript has several limitations but may also inspire potential future directions. One limitation of our first experiment was that the nonlinear regression model did not include random effects to account for repeated measures. Additionally, future work could examine whether the metabolic changes we observed, particularly during backward-force conditions, are accompanied by alterations in stability metrics such as step width or margin of stability. We also acknowledge the limitation that the cart-simulation assumed that the human kinematics remain unchanged. Others have suggested using trajectory optimization to predict the most likely kinematics resulting from human interactions with oscillating systems^39^. We chose to impose fixed kinematics for its simplicity. Based on qualitative observations in the GRF case-study (Fig. 6) it is clear that the participants do in fact change their kinematics, however, it is uncertain whether trajectory optimization would have predicted these changes accurately, because such models typically minimize simplified energetic objectives (e.g., leg work and force rate costs) rather than capturing the broader stability and coordination demands that likely influenced participants’ actual gait.

The limited sample size, particularly in the GRF case study, restricts generalizability, yet the results clearly showed no trend in the effects of spring-stiffness; therefore, future experiments will need to identify the key device improvements rather than simply increasing the sample size. The lack of load cell measurements in the wheeled mass-spring experiment prevents us from knowing exactly how the mass oscillated. Device imperfections related to aspects such as friction and turning do not allow a firm conclusion regarding whether it is truly impossible to assist walking with a wheeled mass-spring system. To address these limitations, future efforts should focus on avoiding arm swing impingement and reducing undesirable forces and friction, potentially by using larger wheels. Future research could also investigate other parameters, such as nonlinear spring characteristics.

The present study shows that although theory and experiments with a robotic tether suggest the feasibility of assisting walking using AP force oscillations, doing so with a passive wheeled mechanism system is challenging. To our best knowledge, this is the first study that investigates the effects of both timed backward and forward forces, and this study also proposes a bidirectional spring mechanism that enables oscillation in a more controlled manner than previous wheeled devices^40^. The finding that the results did not match theoretical predictions is similar to many other examples in wearable robotics, where the results of human experiments did not match predictions^41^. Future iterations may be needed to truly prove or disprove the possibility of assisting walking with AP mass oscillations.

## Supplementary Information

Below is the link to the electronic supplementary material.

Supplementary Material 1

Supplementary Material 2

Supplementary Material 3

## Data Availability

All data generated or analyzed during this study are included in this published article (and its Supplementary Information files).
