# Supplementary material for: Leveraging anteroposterior force oscillations to assist walking
Source: Sci Rep. 2026 Apr 21;16:20342. doi: 10.1038/s41598-026-47823-w (PMC13328586; doi:10.1038/s41598-026-47823-w)
Supplement: Supplementary file 3 — Supplementary Material 3 [file 41598_2026_47823_MOESM3_ESM.zip › Razavi_Supplementary.docx]

**Robotic-tether experiment**

**Table S1.** Overview of desired force profiles by the robotic-tether

| Force profiles | Desired duration (%step) | Desired magnitude (%BW) | Desired forward timing (%step) | Desired backward timing (%step) | # Condition |
| --- | --- | --- | --- | --- | --- |
| Zero-force condition | - | 0 | 0 | 0 | 1 |
| Only forward force conditions | 50 | 7.5 | 15 | - | 2 |
|  |  |  | 40 | - | 3 |
|  |  |  | 65 | - | 4 |
|  |  |  | 90 | - | 5 |
|  |  | 15 | 15 | - | 6 |
|  |  |  | 40 | - | 7 |
|  |  |  | 65 | - | 8 |
|  |  |  | 90 | - | 9 |
| Only backward force | 50 | 7.5 | - | 15 | 10 |
|  |  |  | - | 40 | 11 |
|  |  |  | - | 65 | 12 |
|  |  |  | - | 90 | 13 |
|  |  | 15 | - | 15 | 14 |
|  |  |  | - | 40 | 15 |
|  |  |  | - | 65 | 16 |
|  |  |  | - | 90 | 17 |
| Both forward and backward forces | 50 | 7.5 | 15 | 65 | 18 |
|  |  |  | 40 | 90 | 19 |
|  |  |  | 65 | 15 | 20 |
|  |  |  | 90 | 40 | 21 |
|  |  | 15 | 15 | 65 | 22 |
|  |  |  | 40 | 90 | 23 |
|  |  |  | 65 | 15 | 24 |
|  |  |  | 90 | 40 | 25 |
| No-tether | - | - | - | - | 26 |


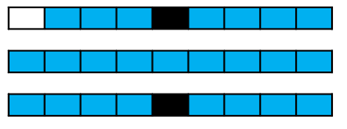


7 min rest

7 min rest

2 min each

No tether

Zero force

Sinusoidal forces (24)

**Figure S1**. Robotic-tether protocol overview.


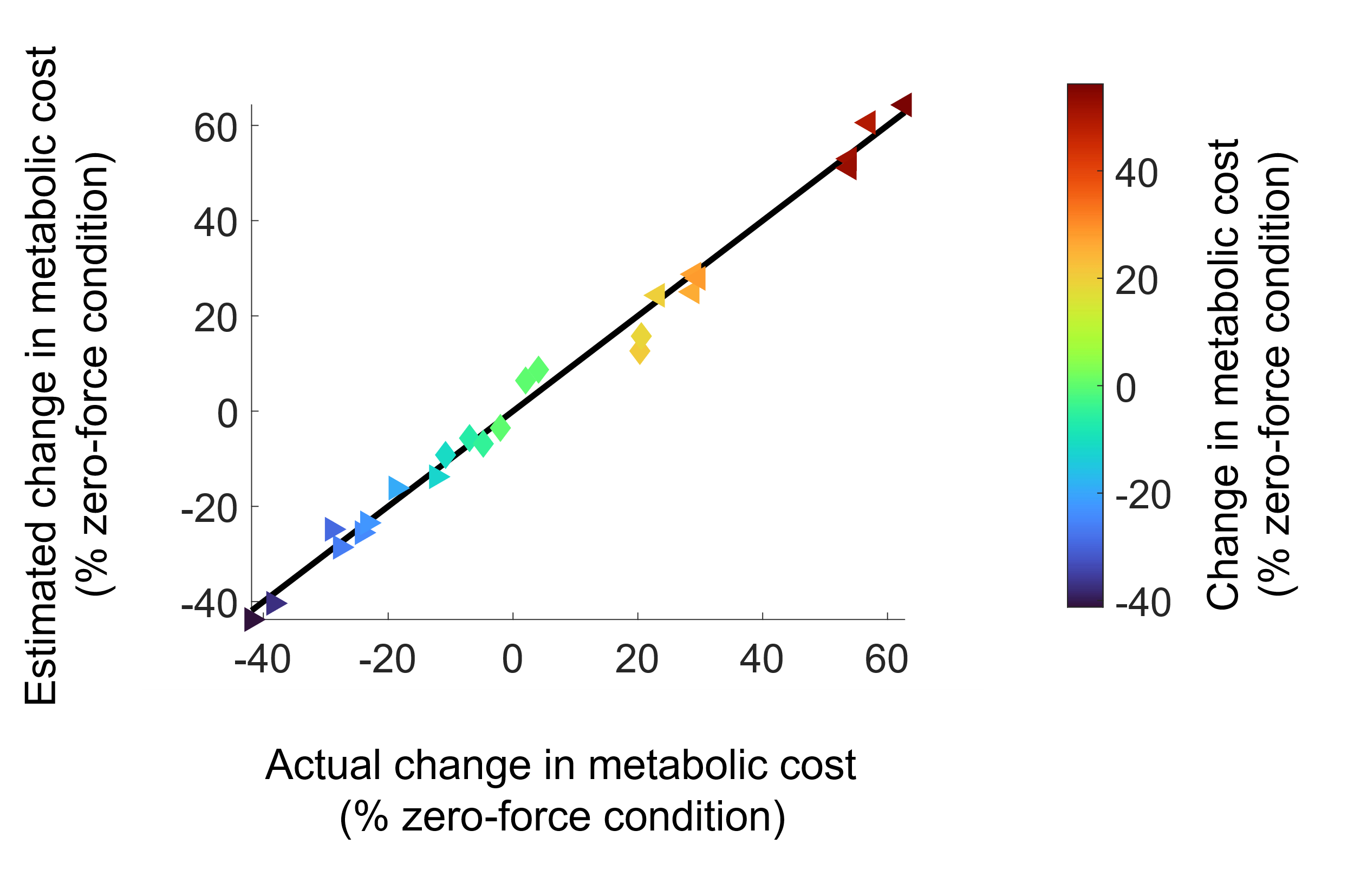


**Figure S2.** Model accuracy. Correlation between the regression-model–estimated change in metabolic cost and the actual change measured across all conditions (R^2^ = 0.99). Individual conditions are shown using forward-facing triangles (forward-only forces), backward-facing triangles (backward-only forces), and diamonds (combined forward and backward forces). Colors represent their relative change in metabolic cost, consistent with Fig. 3.


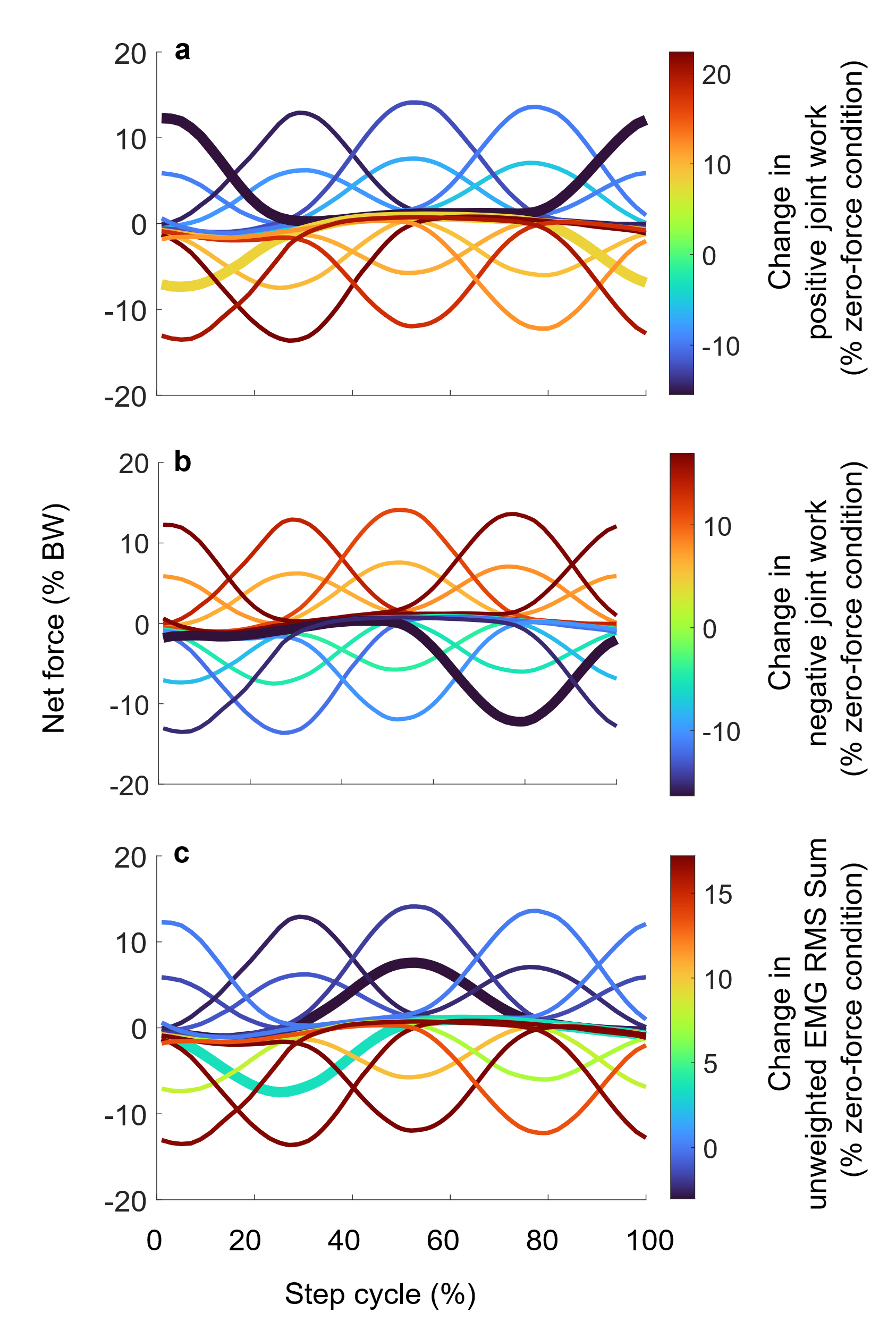


**Figure S3**. Changes in joint work and muscle activation during the robotic-tether experiment in conditions applying only forward or only backward forces. **A**) Applying a forward force during the double stance phase reduced positive joint work. **B**) Applying backward force between 20 to 80% of the step cycle maximally reduced the magnitude of negative joint work (a more positive number indicates a reduced magnitude of negative joint work). **C**) EMG changes did not seem to correspond with the timings of conditions that minimized metabolic cost. This is expected since EMG amplitude reflects neural activation rather than mechanical work or metabolic energy expenditure. For each condition, we computed changes in positive and negative joint work at the hip, knee, and ankle, as well as an unweighted sum of EMG root-mean-square (RMS) activation across lower-limb muscles following the method of Jackson and Collins (2015)^1^, who quantified global muscle demand by computing stride-averaged RMS for each muscle and summing across muscles to obtain a whole-body activation metric.


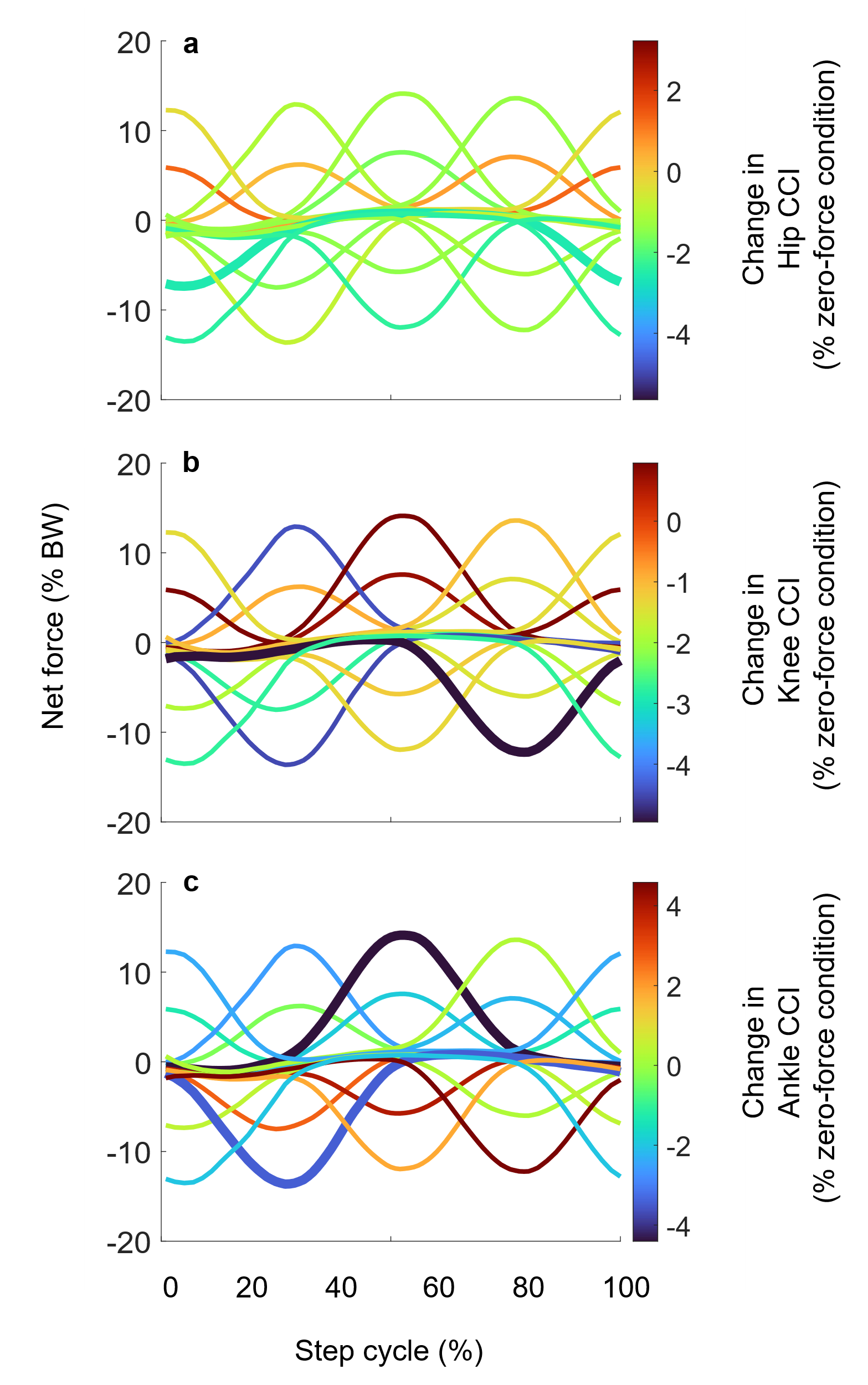


**Figure S4.** Change in mean co-contraction index (CCI) over the step cycle using normalized EMG from (**a**) hip (gluteus maximum, rectus femoris), (**b**) knee (vastus medialis, biceps femoris), and (**c**) ankle (tibialis anterior, medial gastrocnemius) muscle pairs. At each time point, CCI was calculated as the ratio of the lower EMG value to the sum of the two EMG values^2^ and then averaged across the step cycle for each subject and condition.

**Mass-spring experiment**

**Preparatory simulation supplementary method**

All simulations were driven by the measured center of mass kinematic data from the no tether condition of robotic-tether experiment. For each stiffness–damping pair, we performed a forward-dynamics simulation to compute cart acceleration, cart position, and the interaction force between the cart and the person. All forces are in the anteroposterior (AP) direction; forward is defined as positive.

$m_{sub}$ : mass of the subject

$m_{cart}$ : mass of the cart

$x_{sub}$ : AP position of the subject

$x_{cart}$ : AP position of the cart

$v_{sub}$ : AP velocity of the subject

$v_{cart}$ : AP velocity of the cart

$a_{sub}$ : AP acceleration of the subject

$a_{cart}$ : AP acceleration of the cart

$k$: spring stiffness

$d$: damping constant

At each time step, the interaction force was computed as:

| $F_{cart on person}= -k\cdot\left( x_{sub}- x_{cart} \right)- d\cdot\left( v_{sub}- v_{cart} \right)$ | (1) |
| --- | --- |

Cart acceleration followed Newton’s second law:

| $a_{cart}= -\frac{F_{cart on person}}{m_{cart}}$ | (2) |
| --- | --- |

Cart position and velocity were iteratively updated using cart acceleration. The subject’s measured kinematics were used as fixed inputs and did not vary across spring–damper conditions. An initial settling period was removed to ensure that all analyses represented steady, cyclic behavior. For the cart–spring conditions, the AP ground reaction force (GRF) required by the person was computed as:

| $F_{GRF}= m_{sub}\cdot a_{sub}- F_{cart on person}$ | (3) |
| --- | --- |

Two reference conditions were also calculated.

No-cart condition (subject walking alone):

| $F_{GRF_{NoCart}}= m_{sub}\cdot a_{sub}$ | (4) |
| --- | --- |

No-spring (rigid) condition (cart moves with the subject):

| $F_{GRF_{NoSpring}}= \left( m_{sub}+ m_{cart} \right)\cdot a_{sub}$ | (5) |
| --- | --- |

Instantaneous AP mechanical power produced by the subject was:

| $P_{sub}= F_{GRF}\cdot v_{sub}$ | (6) |
| --- | --- |

Positive and negative mechanical power were extracted. Because fluctuations in walking speed were small relative to average speed, the pattern of mechanical power closely followed that of F_GRF_. Metabolic power was estimated using standard efficiency factors (25% for positive work, 120% for negative work):

| $Metabolic = 4\cdot P_{pos}- 1.2\cdot P_{neg}$ | (7) |
| --- | --- |

Stride-normalized metabolic cost was averaged over time.

Percent change relative to the No-Cart condition was computed as:

| $PercentChange =\frac{\left( Metabolic - Metabolic_{NoCart} \right)}{Metabolic_{NoCart}}\cdot100$ | (8) |
| --- | --- |

For consistency across simulations, forces, mechanical power, stiffness, and damping were normalized to % body weight. The resonance frequency of the mass–spring system was calculated after running the forward simulations:

| $f_{\mathrm{resonance}}=\frac{\sqrt{k/m_{cart}}}{2\pi}$ | (9) |
| --- | --- |


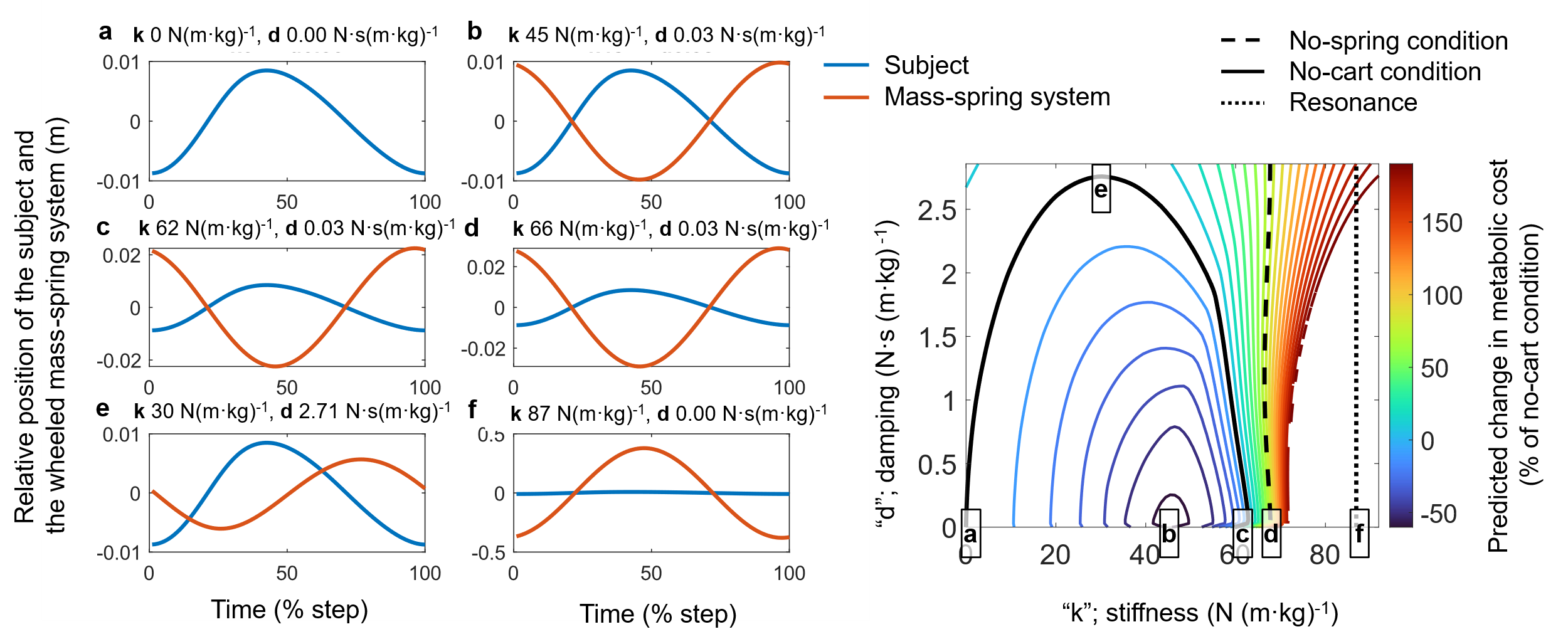


**Figure S5.** Motion between the subject and the wheeled mass–spring system relative to a coordinate system moving at a constant speed. Subplots show relative motion for six combinations of spring stiffness and damping, such that k is stiffness and d is damping. Each subplot (a–f) corresponds to the parameter locations labeled a–f on the stiffness–damping contour plot in Fig. 4.


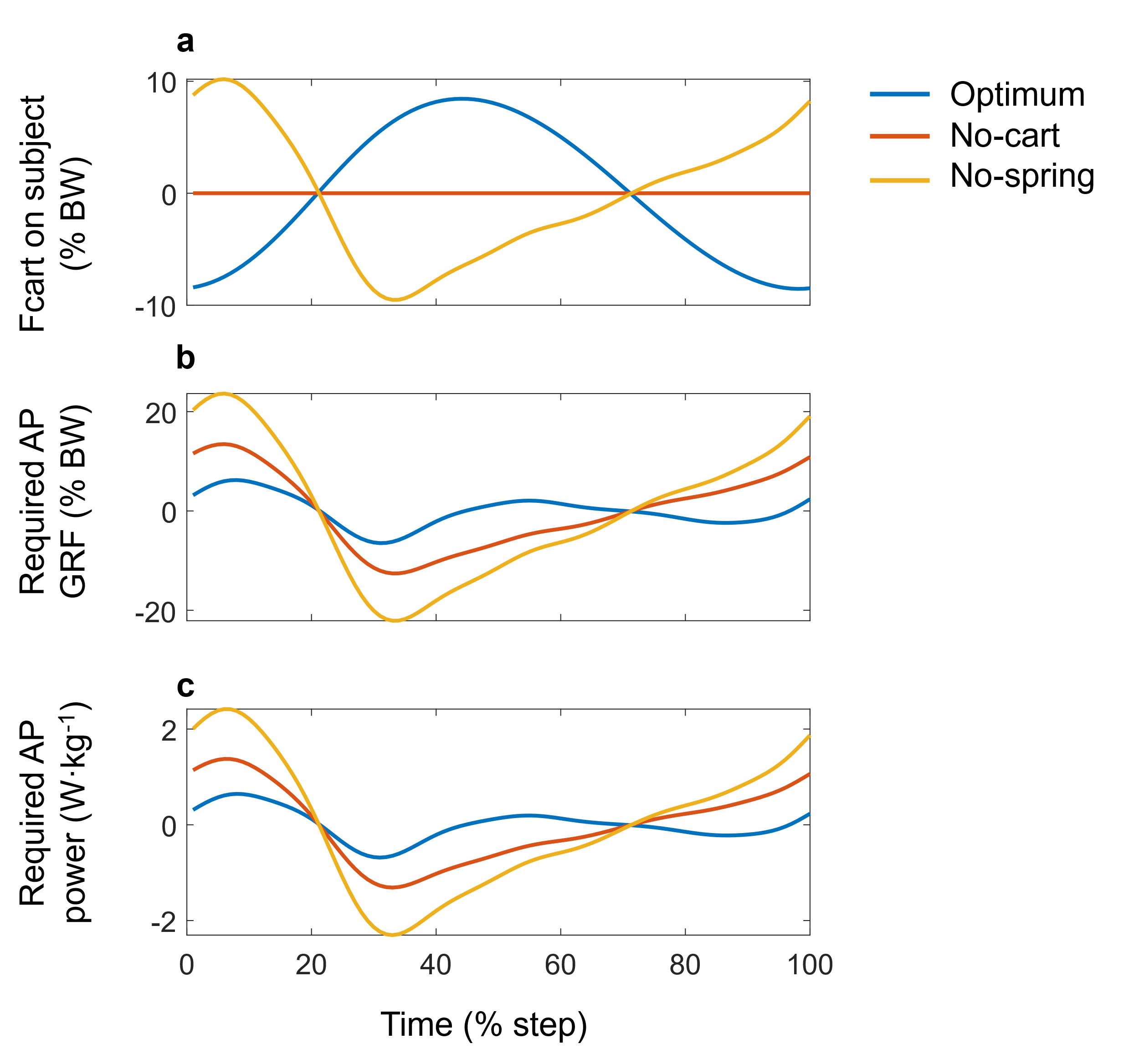


**Figure S6.** A) Force applied to the subject by the wheeled mass–spring system for the optimal spring parameters (blue), the no-spring rigid connection (yellow), and walking with no cart (red). B) Required anteroposterior (AP) ground reaction force (GRF) for the three conditions. C) Required AP mechanical power from the subject. With optimal spring parameters, the cart force oscillates nearly out of phase with the subject’s normal AP dynamics, reducing the required AP GRF and power. In contrast, the no-spring condition produces forces in phase with the subject’s AP GRF, increasing the required GRF and power relative to the no-cart condition.

References

1. Jackson, R. W. & Collins, S. H. An experimental comparison of the relative benefits of work and torque assistance in ankle exoskeletons. *Journal of Applied Physiology* **119**, 541–557 (2015).

2. Chambers, A. J. & Cham, R. Slip-related muscle activation patterns in the stance leg during walking. *Gait & Posture* **25**, 565–572 (2007).
